# Comparison of the efficacy of lamivudine and telbivudine in the treatment of chronic hepatitis B: a systematic review

**DOI:** 10.1186/1743-422X-7-211

**Published:** 2010-09-03

**Authors:** Shushan Zhao, Lanhua Tang, Xuegong Fan, Lizhang Chen, Rongrong Zhou, Xiahong Dai

**Affiliations:** 1 Department of Infectious Diseases, Xiangya Hospital, Central South University, Changsha, China; 2 School of Public Health, Central South University, Changsha, Hunan, China

## Abstract

**Background:**

Chronic viral hepatitis B remains a global public health concern. Currently, several drugs, such as lamivudine and telbivudine, are recommended for treatment of patients with chronic hepatitis B. However, there are no conclusive results on the comparison of the efficacy of lamivudine (LAM) and telbivudine (LdT) in the treatment of chronic hepatitis B.

**Results:**

To evaluate the comparison of the efficacy of LAM and LdT in the treatment of chronic hepatitis B by a systematic review and meta-analysis of clinical trials, we searched PUBMED (from 1990 to April 2010), Web of Science (from 1990 to April 2010), EMBASE (from 1990 to April 2010), CNKI (National Knowledge Infrastructure) (from 1990 to April 2010), VIP database (from 1990 to April 2010), WANFANG database (from 1990 to April 2010), the Cochrane Central Register of Controlled Trials and the Cochrane Database of Systematic Review. At the end of one-year treatment, LdT was better than LAM at the biochemical response, virological response, HBeAg loss, therapeutic response, while less than at the viral breakthrough and viral resistance, but there was no significant difference in the HBeAg seroconversion and HBsAg response. LdT was better than LAM at the HBeAg seroconversion with prolonged treatment to two years.

**Conclusions:**

In summary, LdT was superior in inhibiting HBV replication and preventing drug resistance as compared to LAM for CHB patients. But LdT may cause more nonspecific adverse events and can lead to more CK elevation than LAM. It is thus recommended that the LdT could be used as an option for patients but adverse events, for example CK elevation, must be monitored.

## Background

Chronic hepatitis B virus (HBV) infection is a serious global public health problem associated with cirrhosis, liver failure and hepatocellular carcinoma (HCC) [1]. Of the two billion people who have been infected, more than 350 million have chronic hepatitis[2]. It is estimated that between 235,000 and 328,000 people die annually due to liver cirrhosis and hepatocellular carcinoma, respectively[3]. Currently, several drugs are recommended for treatment of patients with chronic hepatitis B. These drugs can be divided into two main groups based on their mechanism of action, namely immunomodulatory drugs like alpha interferons and anti-viral drugs including lamivudine, adefovir, entecavir, tenofovir, and telbivudine[4].

LdT was approved by the US Food and Drug Administration (FDA) on October 25, 2006. It is an L-nucleoside that is structurally related to lamivudine and highly selective for hepatitis B virus DNA and inhibits viral DNA synthesis with no effect on human DNA or other viruses[5]. In the woodchuck model of HBV infection, viral replication was inhibited within the first few days of treatment and was maintained throughout the treatment period. Then viral rebound with pretreatment levels between week 4 and week 8[5]. A placebo-controlled dose-escalation trial investigated daily dosing levels of LdT between 25 and 800 mg/day for 4 weeks. This study showed that LdT induced striking dose-related suppression of serum HBV DNA levels and a nearly maximal viral load reduction was obtained at dosages of 400-800 mg/day[6]. One-year data from the GLOBE study has recently been presented[7]. Among patients with HBeAg-positive chronic hepatitis B, the rates of HBeAg seroconversion, virological response and HBeAg response were nonsignificantly higher in patients treated with LdT than in patient treated with LAM[7-9]. However other trials did not support this result[10,11]. And recently, some randomized controlled clinical trials compared the efficacy of LAM and LdT in the treatment of chronic hepatitis B and had different results. Thus, we conducted this systematic review of these trials to assess the evidence obtained on the efficacy of LdT treatment in chronic HBV infection.

## Methods

### Search strategy

We searched the following databases until April 2010: PUBMED (from 1990 to April 2010), Web of Science (from 1990 to April 2010), EMBASE (from 1990 to April 2010), CNKI (National Knowledge Infrastructure) (from 1990 to April 2010), VIP database (from 1990 to April 2010), WANFANG database (from 1990 to April 2010), the Cochrane Central Register of Controlled Trials and the Cochrane Database of Systematic Review. Of these databases, CNKI, WANFANG and VIP databases provide literatures in Chinese. The search process was designed to find initially all trials involving terms: "Hepatitis B", "lamivudine", "telbivudine","randomized controlled trial" (and multiple synonyms for each term). Reference lists from retrieved documents were also searched. Computer searches were supplemented with a manual search. Search results were downloaded to a reference database and further screened. Two authors (S. S. Zhao and L. H. Tang) independently screened all citations and abstracts identified by the search strategy to identify potentially eligible studies.

### Types of studies

All relevant randomised clinical trials will be included, irrespective of language, or blinding. Quasi-randomised studies, which use quasi-random method of allocating participants to different interventions, and observational studies will be excluded except for their report on harms.

### Types of participants

Male or female patients, of any age or ethnic origin, who have chronic hepatitis B, defined as chronic hepatitis B virus infection with evidence of hepatitis (alanine aminotransferase (ALT) elevation of at least one and a half times the upper limit of normal range) and of viral replication (detectable hepatitis B virus DNA by DNA hybridisation method or polymerase chain reaction (PCR)), will be included. Patients with cirrhosis, decompensated liver disease, HIV, hepatocellular carcinoma, prior liver transplantation and concomitant renal failure was excluded.

### Types of interventions

The comparisons will include lamivudine versus telbivudine.

### Types of outcome measures

Proportion of patients with biochemical response, virological response, HBeAg seroconversion, HBeAg loss, therapeutic response, HBsAg response, creatine kinase (CK) elevation at the end of one-year treatment or two-year treatment.

### Data extraction

Data was extracted independently by both authors (S. S. Zhao and L. H. Tang) using a pre-designed data extraction form and the information subsequently was entered into Review Manager (RevMan 5.0). Information was extracted on data source; eligibility; methods; participants (age range, exclusion criteria, sample size, gender); interventions; and results. We resolved any discrepancies between the extracted data by discussion, and, if required, referral to the third author (R. R. Zhou). Where data were not clear or not presented by the author in the publication, we attempted to contact the trial author for further details.

### Quality assessment

Quality of the trials was assessed using the QUOROM guidelines as well as using the Jadad scale[12].

### Data analysis

Data analysis was carried out with the use of Review Manager Software 5.0(Cochrane Collaboration, Oxford, United Kingdom). For each eligible study, dichotomous data were presented as relative risk (RR), which is the probability that a member of an exposed group will develop a disease relative to the probability that a member of an unexposed group will develop that same disease, and continuous outcomes were presented as weighted mean difference (WMD), which is calculated as the difference between the mean value in the treatment and control groups, both with 95% confidence intervals (CI). Meta-analysis was performed using fixed-effect or random-effect methods, depending on the absence or presence of significant heterogeneity. Statistical heterogeneity between trials was evaluated by the chi-square and I-square (I^2^) tests, with significance set at P < 0.10. In the absence of statistically significant heterogeneity, the fixed-effect method was used to combine the results. When heterogeneity was confirmed (P < 0.10), the random-effect method was used. Additionally, sensitivity analysis should be carried out if low quality trials were included. The overall effect was tested using z scores calculated by Fisher's z' transformation, with significance set at P < 0.05.

## Results

We searched relevant literatures, and finally a total of 171 studies identified by the searches(PUBMED:8; Web of Science:12; EMBASE:37; CNKI:42; VIP database:18; WANFANG database:33; the Cochrane Central Register of Controlled Trials and the Cochrane Database of Systematic Review:21). By scanning titles and abstracts, 142 redundant publications, review, and meta-analysis were excluded. After referring to full texts, 18 studies that did not satisfy the inclusion criteria were removed from consideration. Eleven studies were left for analysis which involved 2964 patients in total [6-11,13-17], of whom 1475 were included in LAM groups and 1489 were included in LdT groups. According to treatment period, we divided the studies into two subgroups: one-year treatment group[6-11,13,14] and two-year treatment group[15-17]. In addition, all studied populations with comparable baseline characteristics between LAM groups and LdT groups. Of the eleven trials, six were published in English[6,7,10,15-17] and the others were published in Chinese[8,9,11,13,14]. The detailed information of included trials was summarized in table 1 and table 2.

**Table 1:** Description of included randomized controlled trials

Study	Study design	Grade	Treatment options	Study location	dosage of drugs		Treatment
					LAM	LdT	
Lai 2005[6]	RCT, DB	5	LAM vs LdT	Global	100 mg	400/600 mg	12 months
Lai 2007[7]	RCT, DB	5	LAM vs LdT	Global	100 mg	600 mg	12 months
Rasenack 2007[17]	RCT, DB	4	LAM vs LdT	Global	100 mg	600 mg	24 months
Jia 2007[16]	RCT, DB	4	LAM vs LdT	China	100 mg	600 mg	24 months
Hou 2008[10]	RCT, DB	4	LAM vs LdT	China	100 mg	600 mg	12 months
Liaw 2009[15]	RCT, DB	5	LAM vs LdT	Global	100 mg	600 mg	24 months
Cai 2009[13]	RCT, DB	5	LAM vs LdT	China	100 mg	600 mg	12 months
Yang 2009[14]	RCT	3	LAM vs LdT	China	100 mg	600 mg	12 months
Zhong 2009[11]	RCT	3	LAM vs LdT	China	100 mg	600 mg	12 months
Chen 2009[9]	N/A	2	LAM vs LdT	China	100 mg	600 mg	12 months
Tang 2009[8]	RCT	3	LAM vs LdT	China	100 mg	600 mg	12 months

RCT, randomized controlled trial; DB, double blind; LAM, Lamivudine; LdT, Telbivudine

**Table 2:** Characteristics of included clinical trials in systematic review

Study	Entry e status	Sample size (n)	Sex		Median (range) age (y)		Mean (range) weight (kg)		Intervention	
			Male	Female	LAM	LdT	LAM	LdT	LAM	LdT
Lai 2005[6]	HBeAg+	63	49	14	34 (18-61)	40 (19-60)	69 (45-86)	70 (53-120)	19	44
	HBeAg-	N/A	N/A	N/A	N/A	41 (22-68)	N/A	70 (51-96)	N/A	N/A
Lai 2007[7]	HBeAg+	921	684	237	33 (16-67)	32 (16-63)	68 (38-150)	66 (38-126)	463	458
	HBeAg-	446	351	95	43 (18-68)	43 (17-68)	71 (45-148)	72 (42-123)	224	222
Rasenack 2007[17]	HBeAg+	580	N/A	N/A	N/A	N/A	N/A	N/A	289	291
Jia 2007[16]	HBeAg+	290	225	65	29 (15-63)	28 (16-64)	62 (42-96)	62 (43-93)	143	147
	HBeAg-	42	36	6	36 (19-58)	38 (20-56)	65 (49-93)	64 (52-99)	22	20
Hou 2008[10]	HBeAg+	290	225	65	29 (15-63)	28 (16-64)	62 (42-96)	62 (43-93)	143	147
	HBeAg-	42	36	6	36 (19-58)	38 (20-56)	65 (49-93)	64 (52-99)	22	20
Liaw 2009[15]	HBeAg+	921	684	237	33 (16-67)	32 (16-63)	68 (38-150)	66 (38-126)	463	458
	HBeAg-	446	351	95	43 (18-68)	43 (17-68)	71 (45-148)	72 (42-123)	224	222
Cai 2009[13]	HBeAg+	36	34	11	33.62 ± 11.17	29.59 ± 10.17	N/A	N/A	19	17
	HBeAg-	9					N/A	N/A	4	5
Yang 2009[14]	HBeAg+	40	62	38	47.9 ± 8.6		N/A	N/A	50	50
	HBeAg-	60					N/A	N/A		
Zhong 2009[11]	HBeAg+	120	81	39	30 ± 8.5	29 ± 8.2	61 ± 13.6	62 ± 14.2	60	60
	HBeAg-	120	72	48	42 ± 9.5	41 ± 10.1	64 ± 15.2	63 ± 14.5	60	60
Chen 2009[9]	HBeAg+	73	62	11	27.9(16-46)		N/A	N/A	43	30
Tang 2009[8]	HBeAg+	108	N/A	N/A	N/A	N/A	N/A	N/A	52	56
	HBeAg-	56	N/A	N/A	N/A	N/A	N/A	N/A	27	29

### Biochemical response

#### One-year treatment group

**Figure 1:**
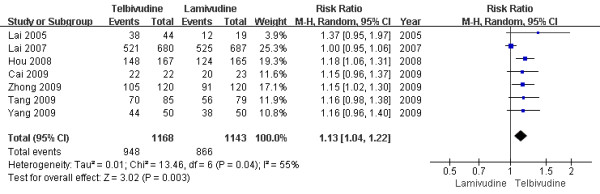
**Effect of telbivudine vs. lamivudine at the end of one-year treatment on Biochemical response**.

Only seven trials[6-8,10,11,13,14] demonstrated the biochemical response rate in this subgroup. According to chi-squared statistic and I square (I^2^), heterogeneity was assessed and had significant differences[Tau^2 ^= 0.01; Chi^2 ^= 13.46, df = 6 (P = 0.04); I^2 ^= 55%]. A summary estimate of the relative risk of LdT versus LAM by use of a random-effects approach. The results of the seven trials showed normalization rates for ALT in the LdT group as 81.2%, compared to 75.8% in the LAM group after one-year treatment. And the biochemical response rates in LdT group was higher than LAM group[RR = 1.13, 95%CI(1.04-1.22), P = 0.003](Figure 1). When a study[7] was removed, the heterogeneity was assessed and not found to be a concern[Chi^2 ^= 0.88, df = 5 (P = 0.97); I^2 ^= 0%]. The difference in response rate between two group were still significantly by use a fixed effects model[87.5% vs. 74.8%, RR = 1.17, 95%CI (1.10-1.25), P < 0.00001].

#### Two-year treatment group

**Figure 2:**
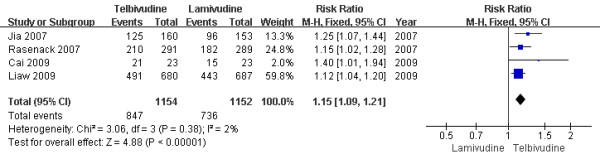
**Effect of telbivudine vs. lamivudine at the end of two-year treatment on Biochemical response**.

Only four trials[13,15-17] demonstrated the biochemical response rate in this subgroup. According to chi-squared statistic and I square (I^2 ^), heterogeneity was assessed and not found to be a concern[Chi^2 ^= 3.06, df = 3 (P = 0.38); I^2 ^= 2%]. The biochemical response rates in LdT group was higher as compared with that in LAM group [73.4% vs. 63.9%, RR = 1.15, 95%CI (1.09-1.21), P < 0.00001] (Figure 2). Additionally, when low-quality study[13] was removed, the difference in response rate was still statistically significantly[73.0% vs. 63.9%, RR = 1.14, 95%CI (1.08-1.21), P < 0.00001].

### Virological response

#### One-year treatment group

**Figure 3:**
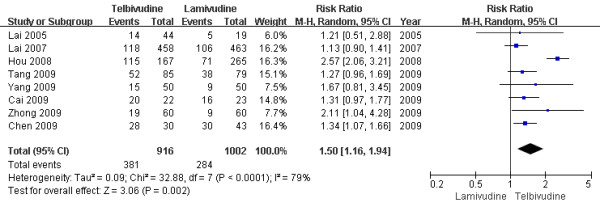
**Effect of telbivudine vs. lamivudine at the end of one-year treatment on Virological response**.

Eight trials[6-11,13,14] demonstrated the virological response rate in this subgroup. According to chi-squared statistic and I square (I^2^), heterogeneity was assessed and had significant differences[Tau^2 ^= 0.09; Chi^2 ^= 32.88, df = 7 (P < 0.0001); I^2 ^= 79%]. A summary estimate of the relative risk of LdT versus LAM by use of a random-effects approach. The results of the eight trials showed virological response rate in the LdT group as 41.6%, compared to 28.3% in the LAM group after one-year treatment. And the virological response rates in LdT group was higher than LAM group[RR = 1.50, 95%CI(1.16-1.94), P = 0.002](Figure 3). When a study[10] was removed, the heterogeneity was assessed and not found to be a concern[Chi^2 ^= 3.91, df = 6 (P = 0.69); I^2 ^= 0%]. The difference in response rate between two group were still significantly by use a fixed effects model[35.5% vs. 28.9%, RR = 1.26, 95%CI (1.10-1.45), P = 0.001].

#### Two-year treatment group

**Figure 4:**
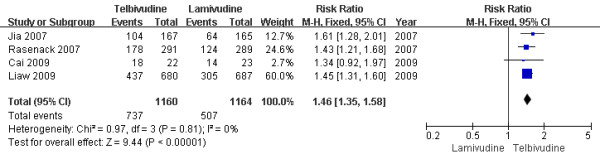
**Effect of telbivudine vs. lamivudine at the end of two-year treatment on Virological response**.

Four trials[13,15-17] demonstrated the virological response rate in this subgroup. According to chi-squared statistic and I square (I^2 ^), heterogeneity was assessed and not found to be a concern[Chi^2 ^= 0.97, df = 3 (P = 0.81); I^2 ^= 0%], allowing use of the fixed effect model for meta-analysis. The results of the four studies showed the virological response rate for the LdT group was 63.5%, while the LAM group response rate was 43.6%. The difference of virological response rates at the end of two years between the two group was statistically significant[RR = 1.46, 95%CI (1.35-1.58), P < 0.00001] (Figure 4). Additionally, when a study[16] was removed, the difference in response rate was still statistically significantly[63.7% vs. 44.3%, RR = 1.44, 95%CI (1.32-1.56), P < 0.00001]. So compared to the LAM group, LdT group was more effective as measured by virological response.

### HBeAg seroconversion

#### One-year treatment group

**Figure 5:**
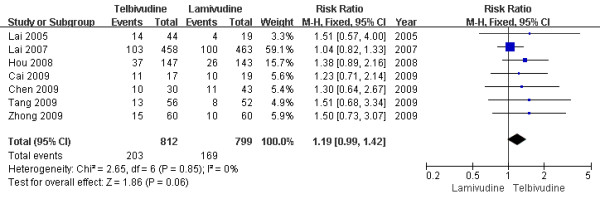
**Effect of telbivudine vs. lamivudine at the end of one-year treatment on HBeAg seroconversion**.

Seven[6-8,10,11,13,14] trials demonstrated the HBeAg seroconversion rate in this subgroup. According to chi-squared statistic and I square (I^2 ^), heterogeneity was assessed and not found to be a concern[Chi^2 ^= 2.65, df = 6 (P = 0.85); I^2 ^= 0%], allowing use of the fixed effect model for meta-analysis. The results of the seven studies showed the virological response rate for the LdT group was 25.0%, while the LAM group response rate was 21.2%. The difference of HBeAg seroconversion rates at the end of one year between the two group was similar[RR = 1.19, 95%CI (0.99-1.42), P = 0.06] (Figure 5). Moreover, when low-quality study[9] was removed, the difference in HBeAg seroconversion rate was still no statistically significant[24.7% vs. 20.9%, RR = 1.44, 95%CI (1.32-1.56), P < 0.00001].

#### Two-year treatment group

**Figure 6:**
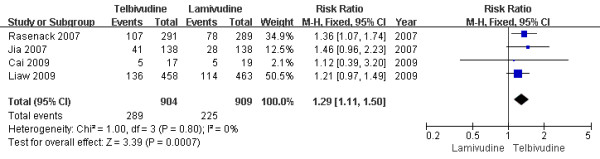
**Effect of telbivudine vs. lamivudine at the end of two-year treatment on HBeAg seroconversion**.

Four trials[13,15-17] demonstrated the HBeAg seroconversion rate in this subgroup. According to chi-squared statistic and I square (I^2 ^), heterogeneity was assessed and not found to be a concern[Chi^2 ^= 1.00, df = 3 (P = 0.80); I^2 ^= 0%]. The results of the four studies showed the HBeAg seroconversion rate for the LdT group was 32.0%, while the LAM group response rate was 24.8%. The difference of HBeAg seroconversion rates at the end of two years between the two group was statistically significant[RR = 1.29, 95%CI (1.11-1.50), P < 0.0007] (Figure 6). Additionally, when a study[17] was removed, the difference in HBeAg seroconversion rate was still statistically significantly[29.7% vs. 23.7%, RR = 1.25, 95%CI (1.04-1.51), P = 0.02]. So LdT group was similar with LAM group with respect to seroconversion of HBeAg after one year treatment, but more effective at the end of two years treatment.

### HBeAg loss

#### One-year treatment group

**Figure 7:**
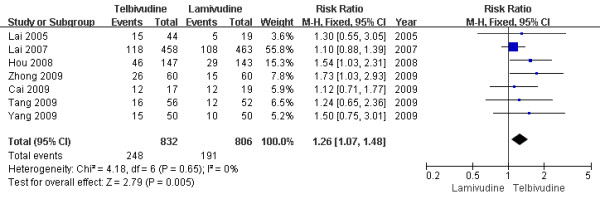
**Effect of telbivudine vs. lamivudine at the end of one-year treatment on HBeAg loss**.

The rate of HBeAg loss at the end of the one-year treatment is shown in Figure 7. The results of the seven studies[6-11,13] showed the HBeAg loss rate of LdT group was 29.8%, while the LAM group rate was 23.7%. There was on statistical heterogeneity(Chi^2 ^= 4.18, df = 6 (P = 0.65); I^2 ^= 0%), and fixed effect model was used. The difference of the HBeAg loss rates at the end of the one-year treatment between the two group achieved statistical significance[RR = 1.26, 95%CI (1.07-1.48), P = 0.005] (Figure 7). Additionally, when low-quality study[11] was removed, the difference in HBeAg loss rate was still statistically significantly[28.8% vs. 23.6%, RR = 1.22, 95%CI (1.03-1.44), P = 0.02].

#### Two-year treatment group

**Figure 8:**
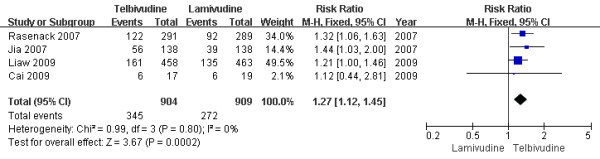
**Effect of telbivudine vs. lamivudine at the end of two-year treatment on HBeAg loss**.

According to chi-squared statistic and I square (I^2 ^), heterogeneity was assessed and not found to be a concern[Chi^2 ^= 0.99, df = 3 (P = 0.80); I^2 ^= 0%]. The results of the four studies[13,15-17] showed the HBeAg loss rate for the LdT group was 38.1%, while the LAM group response rate was 29.9%. The difference of HBeAg loss rates at the end of two years between the two group was statistically significant[RR = 1.27, 95%CI (1.12-1.45), P = 0.0002] (Figure 8). Additionally, when a effective study[17] was removed, the difference in HBeAg loss rate was still statistically significantly[36.47% vs. 29.0%, RR = 1.25, 95%CI (1.07-1.47), P = 0.006].

### Therapeutic response

#### One-year treatment group

**Figure 9:**
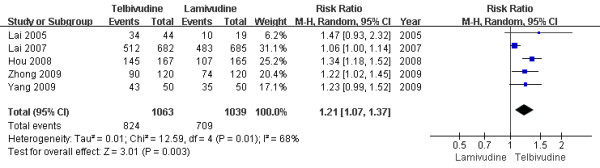
**Effect of telbivudine vs. lamivudine at the end of one-year treatment on Therapeutic response**.

Only five trials[6,7,10,11,14] demonstrated the therapeutic response rate in this subgroup. According to chi-squared statistic and I square (I^2^), heterogeneity was assessed and had significant differences[Tau^2 ^= 0.01; Chi^2 ^= 12.59, df = 4 (P = 0.01); I^2 ^= 68%]. A summary estimate of the relative risk of LdT versus LAM by use of a random-effects approach. The results of the five trials showed therapeutic response rates in the LdT group as 77.5%, compared to 68.2% in the LAM group after one-year treatment. And the therapeutic response rates in LdT group was higher than LAM group[RR = 1.21, 95%CI(1.07-1.37), P = 0.003] (Figure 9). When low-quality study[11] was removed, the heterogeneity was assessed and was still a concern[Tau^2 ^= 0.02; Chi^2 ^= 11.95, df = 3 (P = 0.008); I^2 ^= 75%]. The difference in response rate between two group were still significantly by use a random-effects model[77.8% vs. 69.1%, RR = 1.22, 95%CI (1.04-1.43), P < 0.01].

#### Two-year treatment group

**Figure 10:**
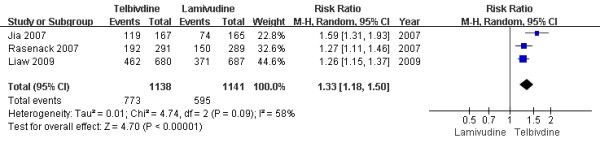
**Effect of telbivudine vs. lamivudine at the end of two-year treatment on Therapeutic response**.

According to chi-squared statistic and I square (I^2 ^), heterogeneity was assessed and had significant differences [Chi^2 ^= 4.74, df = 2 (P = 0.09); I^2 ^= 58%]. The results of the three studies[15-17] showed the therapeutic response rate for the LdT group was 67.9%, while the LAM group response rate was 52.1%. The difference of therapeutic response rates at the end of two years between the two group was statistically significant[RR = 1.33, 95%CI (1.18-1.50), P < 0.00001] (Figure 10). Additionally, when a effective study[17] was removed, the difference in HBeAg loss rate was still statistically significantly[67.4% vs. 53.3%, RR = 1.26, 95%CI (1.17-1.36), P < 0.00001].

### HBsAg response

Of the eleven included studies, only two studies[13,15] detected serum HBsAg. One study[15] reported the HBsAg response at the end of one-year treatment while the other[13] reported the HBsAg response at the both end of treatment. The results of the study showed the HBsAg response rate for LdT group was 4.5%, while the LAM group response rate was 4.3% after one-year treatment. The difference of HBsAg response rates between the two group was similar[RR = 0.96, 95%CI (0.06-14.37), P = 0.97]. Two studies reported the HBsAg response rates, but no statistically significant difference were seen between LdT group and LAM group[1.3% vs. 1.1%, RR = 1.11, 95%CI (0.43-2.85), P = 0.83].

### Safety

Four studies[6,7,10,11] reported the viral breakthrough rate during the one year treatment. The results of the study showed the viral breakthrough rates for LdT group and LAM group were 4.8% and 14.8% respectively. The difference was statistically significantly[RR = 0.33, 95%CI (0.24-0.45), P < 0.00001]. Only one study[15] showed the viral breakthrough rate at the end of two-year treatment, which said the the viral breakthrough rate for the LdT group was 24.9%, while the LAM group response rate was 41.2%. The difference was statistically significantly[RR = 0.59, 95%CI (0.51-0.69), P < 0.00001].

Four studies[7,8,10,11] reported the viral resistance rate during the one year treatment. The results of the study showed the viral resistance rates for LdT group and LAM group were 5.2% and 12.8% respectively. The difference was statistically significantly[RR = 0.41, 95%CI (0.30-0.55), P < 0.00001]. Only one study[15] showed the viral resistance rate at the end of two-year treatment, which said the the viral resistance rate for the LdT group was 20.4%, while the LAM group response rate was 35.1%. The difference was statistically significantly[RR = 0.58, 95%CI (0.49-0.70), P < 0.00001].

**Figure 11:**
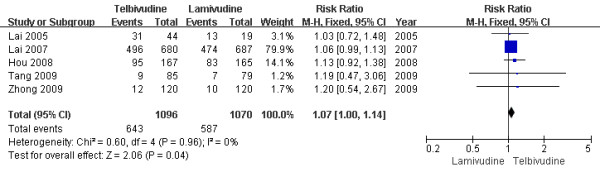
**Effect of telbivudine vs. lamivudine at the end of one-year treatment on adverse events**.

Patients reported nonspecific symptoms such as fatigue, cough, headache, upper respiratory tract infection. Five studies reported[6-8,10,11] the adverse events rate at the end of one-year treatment. The result of the study were statistically significantly[RR = 1.07, 95%CI (1.00-1.14), P = 0.04] (Figure 11). And, even when two low-quality studies[8,11] were removed, the difference between two groups still statistically significantly. However one study reporting the adverse events rate at the end of two-year treatment showed the result were similar[RR = 1.05, 95%CI (1.00-1.11), P = 0.07]. So it is interesting results and hard to say whether LdT can cause more adverse events or not.

### Creatine kinase (CK) elevation

**Figure 12:**
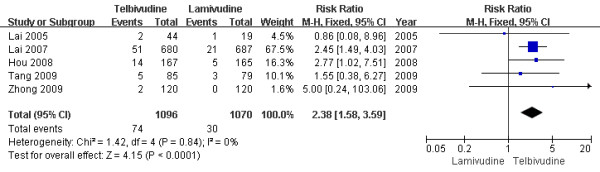
**Effect of telbivudine vs. lamivudine at the end of one-year treatment on CK elevation**.

**Figure 13:**
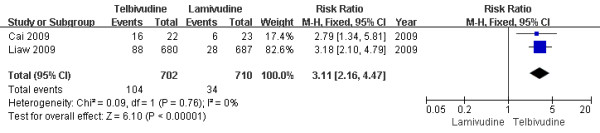
**Effect of telbivudine vs. lamivudine at the end of two-year treatment on CK elevation**.

Five studies[6-8,10,11] reported Grade 3 or 4 CK elevations rate at the end of one-year treatment. According to chi-squared statistic and I square (I^2 ^), heterogeneity was assessed and not found to be a concern[Chi^2 ^= 1.42, df = 4 (P = 0.84); I^2 ^= 0%]. The difference of CK elevations rates between the two group was statistically significant[6.8% vs. 2.8%, RR = 2.38, 95%CI (1.58-3.59), P < 0.0001] (Figure 12). when an effective study[7] or low-quality[8,11] was removed, the difference in CK elevations rate was still statistically significantly. Two studies[13,15] reported Grade 3 or 4 CK elevations at the end of two-year treatment. The heterogeneity was not a concern, and the difference of CK elevation rates between the two group was statistically significant[14.8% vs. 4.8%, RR = 3.11, 95%CI (2.16-4.47), P < 0.0001] (Figure 13). So increased CK occurred more frequently during telbivudine treatment during clinical trials.

## Discussion

Although new approved powerful agents like entecavir and tenofovir are available now in certain countries, there are challenges ahead to be used widely. First, the prevalence of chronic HBV infection varies greatly in different parts of the world. Based on the prevalence of HBV surface antigen(HBsAg) carrier rate in the general population, sub-Saharan African, East Asian and Alaskan populations are classified as having high HBV endemicity[18] (HBsAg carriage > 8%). However the majority of countries in those areas have low-income economies, and the infrastructure of the healthcare system is not satisfactory. There are limitations in the reimbursement of anti-HBV therapy, either in the selection of agent or the duration of dosing. Therefore, lamivudine and telbivudine with low costs are still widely used[19]. Second, tenofovir is a new approved agents which hasn't been introduced to lots of low-income economy countries like China. So lamivudine and telbivudine are more widely used in treatment of CHB.

**Figure 14:**
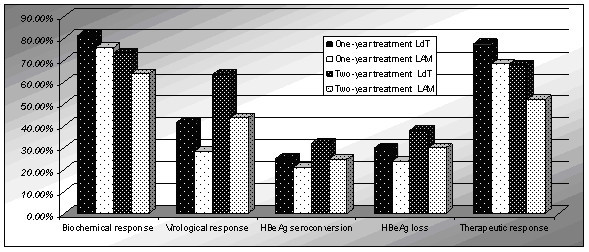
**End of one-years and two-years in biochemical response, virological response, HBeAg seroconversion, HBeAg loss, therapeutic response**.

**Figure 15:**
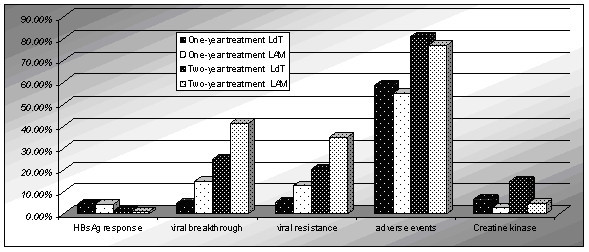
**End of one-years and two-years in HBsAg response, viral breakthrough, viral resistance, adverse events and creatine kinase**.

In this systematic review, we focus on the comparison of the efficay of lamivudine and telbivudine in the treatment of CHB. The results showed that at the end of one-year treatment, LdT was better than LAM at the biochemical response, virological response, HBeAg loss, therapeutic response, while less than at the viral breakthrough and viral resistance, but there was no significant difference in the HBeAg seroconversion and HBsAg response. However, the difference between one-year treatment and two-year treatment was that LdT was better than LAM at the HBeAg seroconversion. So the rate of HBeAg seroconversion increased with prolonged treatment significantly. The result of this systematic review showed telbivudine had greater antiviral efficacy than did lamivudine. Nonetheless the rate of virological response, HBeAg loss, viral breakthrough, viral resistance, adverse events and creatine kinase increased while the biochemical response, therapeutic response and HBsAg response decreased with prolonged treatment(Figure 14, Figure 15). Particular attention should be paid to the adverse events. This systematic review indicated that the frequencies of adverse events were more for patients who received telbivudine than for those who received lamivudine, and increased with the prolonged treatment. Especially, Grade 3 or 4 increased CK occurred more frequently during telbivudine treatment. The RR was 3.11 and 95% CI was between 2.16 and 4.47. In contrast, LAM is more tolerable than LdT and has fewer side effects.

The limitations of the systematic review warrant some discussion. First, the methodological of the trials has limitations. Some studies were not double-blinded. The lack of blinding could affect the outcomes assessed[20]. Even one study[9] didn't perform at random which can lead selection bias[21]. Second, the potentially important limitation of systematic review is publication bias, the fact that not all research is published. Compared to positive studies, negative studies may be less likely to be published and more likely to take longer to be published, which can affect the validity of meta-analysis in this review[22]. Besides only publish in English and Chinese studies were included in this systematic review which may cause language bias. The manual search of many medical journals published in different languages will help to reduce this bias[23]. Additional issues include small trial sizes and a high rate of studies that were conducted in China.

In summary, LdT was superior in inhibiting HBV replication and preventing drug resistance as compared to LAM for CHB patients. But LdT may occur more nonspecific adverse events and can lead more CK elevation than LAM. It is thus recommended that the LdT could be used as an option for patients but adverse events, for example CK elevation, must be monitored. More high-quality, well-designed, randomized controlled, multi-center trails that are adequately powered are clearly needed to guide evolving standards of care for CHB.

## Competing interests

The funding source had no influence on study design, in the collection, analysis, and interpretation of the data, in the writing of the manuscript, or in the decision to submit the manuscript for publication. The contents are solely the responsibility of the authors and do not necessarily represent the views of the funding source.

## Authors' contributions

XGF conceived the study, provided fund supporting and revised the manuscript critically for important intellectual content. SSZ, RRZ and LHT made substantial contributions to its design, acquisition, analysis and interpretation of data. LZC, and XHD, participated in the design, acquisition, analysis and interpretation of data. All authors contributed equally to this manuscript. All authors read and approved the final manuscript.

## References

[B1] Safioleas M Lygidakis NJ Manti C Hepatitis B today Hepatogastroenterology 2007 54 545 548 17523319

[B2] Poland GA Jacobson RM Clinical practice: prevention of hepatitis B with the hepatitis B vaccine N Engl J Med 2004 351 2832 2838 10.1056/NEJMcp041507 15625334

[B3] Perz JF Armstrong GL Farrington LA Hutin YJ Bell BP The contributions of hepatitis B virus and hepatitis C virus infections to cirrhosis and primary liver cancer worldwide J Hepatol 2006 45 529 538 10.1016/j.jhep.2006.05.013 16879891

[B4] Nash K Telbivudine in the treatment of chronic hepatitis B Adv Ther 2009 26 155 169 10.1007/s12325-009-0004-y 19225726

[B5] Bryant ML Bridges EG Placidi L Faraj A Loi AG Pierra C Dukhan D Gosselin G Imbach JL Hernandez B Antiviral L-nucleosides specific for hepatitis B virus infection Antimicrob Agents Chemother 2001 45 229 235 10.1128/AAC.45.1.229-235.2001 11120971 PMC90266

[B6] Lai CL Leung N Teo EK Tong M Wong F Hann HW Han S Poynard T Myers M Chao G A 1-year trial of telbivudine, lamivudine, and the combination in patients with hepatitis B e antigen-positive chronic hepatitis B Gastroenterology 2005 129 528 536 16083710 10.1016/j.gastro.2005.05.053

[B7] Lai CL Gane E Liaw YF Hsu CW Thongsawat S Wang Y Chen Y Heathcote EJ Rasenack J Bzowej N Telbivudine versus lamivudine in patients with chronic hepatitis B N Engl J Med 2007 357 2576 2588 10.1056/NEJMoa066422 18094378

[B8] Tang DC Xie DM Xu JJ He JY Yao LH Yang ZZ Li YF Clinical Study on Telbivudine Treating Chronic Hepatitis B Journal of Zhejiang University of Traditional Chinese Medicine 2009 33 214 215

[B9] Chen SX Hu XZ Zhu F Wan B Liu P Therapeutic effect of 48 weeks of telbivudine on HBeAg-positive chronic hepatitis B Infect Dis Info 2009 22 216 218

[B10] Hou J Yin YK Xu D Tan D Niu J Zhou X Wang Y Zhu L He Y Ren H Telbivudine versus lamivudine in Chinese patients with chronic hepatitis B: Results at 1 year of a randomized, double-blind trial Hepatology 2008 47 447 454 10.1002/hep.22075 18080339

[B11] Zhong XH Liu Y Xu C Xu LM Wang M Li MZ The study of efficacy and side effects of telbivudine and lamivudine for treatment of patients with chronic hepatitis B Chinese Journal of Integrated Traditional and Western Medicine on Liver Diseases 2009 19 19 21

[B12] Jadad AR Moore RA Carroll D Jenkinson C Reynolds DJ Gavaghan DJ McQuay HJ Assessing the quality of reports of randomized clinical trials: is blinding necessary? Control Clin Trials 1996 17 1 12 10.1016/0197-2456(95)00134-4 8721797

[B13] Cai W Efficacy and its influent factors of telbivudine or lamivudine treated with chronic hepatitis B patients Shanghai Jiao Tong University school of medicine 2009

[B14] Yang JN A comparison of curative effect between telbivudine and lamivudine in patients with chronic hepatitis B virus Chin J Prim Med Pharm 2009 16 1193 1194

[B15] Liaw YF Gane E Leung N Zeuzem S Wang Y Lai CL Heathcote EJ Manns M Bzowej N Niu J 2-Year GLOBE trial results: telbivudine Is superior to lamivudine in patients with chronic hepatitis B Gastroenterology 2009 136 486 495 10.1053/j.gastro.2008.10.026 19027013

[B16] Jia JD Hou JL Yin YK Xu DZ Tan D Niu J Zhou XQ Wang Y Zhu L Brown N Two-year results of a phase III comparative trial of telbivudine vs lamivudine in chinese patients Journal of Hepatology 2007 46 S189 S189 10.1016/S0168-8278(07)62095-9

[B17] Rasenack J Poynard T Lai CL Gane E Brown NA Heathcote J Efficacy of telbivudine vs lamivudine at 2 years in patinents with HbeAg-positive chronic hepatitis B who are eligible for treatment based on guidelines Journal of Hepatology 2007 46 S195 S195 10.1016/S0168-8278(07)62111-4

[B18] Lavanchy D Hepatitis B virus epidemiology, disease burden, treatment, and current and emerging prevention and control measures J Viral Hepat 2004 11 97 107 10.1046/j.1365-2893.2003.00487.x 14996343

[B19] Liaw YF Antiviral therapy of chronic hepatitis B: opportunities and challenges in Asia J Hepatol 2009 51 403 410 10.1016/j.jhep.2009.04.003 19467727

[B20] Juni P Altman DG Egger M Systematic reviews in health care: Assessing the quality of controlled clinical trials BMJ 2001 323 42 46 10.1136/bmj.323.7303.42 11440947 PMC1120670

[B21] Bauer P Koenig F Brannath W Posch M Selection and bias--two hostile brothers Stat Med 2010 29 1 13 19844944 10.1002/sim.3716

[B22] Thornton A Lee P Publication bias in meta-analysis: its causes and consequences J Clin Epidemiol 2000 53 207 216 10.1016/S0895-4356(99)00161-4 10729693

[B23] Egger M Zellweger-Zahner T Schneider M Junker C Lengeler C Antes G Language bias in randomised controlled trials published in English and German Lancet 1997 350 326 329 10.1016/S0140-6736(97)02419-7 9251637

